# IL-1R and Inflammasomes Mediate Early Pulmonary Protective Mechanisms in Respiratory *Brucella Abortus* Infection

**DOI:** 10.3389/fcimb.2018.00391

**Published:** 2018-11-05

**Authors:** M. Soledad Hielpos, Andrea G. Fernández, Juliana Falivene, Iván M. Alonso Paiva, Florencia Muñoz González, Mariana C. Ferrero, Priscila C. Campos, Angelica T. Vieira, Sergio Costa Oliveira, Pablo C. Baldi

**Affiliations:** ^1^Facultad de Farmacia y Bioquímica, Cátedra de Inmunología, Universidad de Buenos Aires, Buenos Aires, Argentina; ^2^CONICET-Universidad de Buenos Aires, Instituto de Estudios de la Inmunidad Humoral, Buenos Aires, Argentina; ^3^Department of Biochemistry and Immunology, Institute of Biological Sciences, Federal University of Minas Gerais, Belo Horizonte, Brazil

**Keywords:** *Brucella abortus*, respiratory infection, innate immunity, IL-1β, inflammasomes

## Abstract

*Brucella* spp. infection is frequently acquired through contaminated aerosols. The role of interleukin-1 beta (IL-1β) in the early pulmonary response to respiratory *Brucella* infection is unknown. As shown here, IL-1β levels in lung homogenates and bronchoalveolar lavage fluid (BALF) of mice intratracheally inoculated with *B. abortus* were increased at 3 and 7 days p.i. At 7 days p.i., pulmonary CFU numbers were higher in IL-1 receptor (IL-1R) knockout (KO) mice than in wild type (WT) mice. At different times p.i. CFU in lungs and BALF were higher in mice lacking some inflammasome components (caspase-1, AIM2, NLRP3) than in WT mice. At 2 days p.i. pulmonary levels of IL-1β and CXCL1 (neutrophils chemoattractant) were lower in caspase-1/11 KO mice. At day 3 p.i., neutrophils counts in BALF were lower in caspase-1/11 KO mice than in WT mice. During *in vitro* infections, IL-1β secretion was lower in alveolar macrophages from caspase-1/11, NLRP3 or AIM2 KO mice than in WT controls. Similarly, IL-1β production by *B. abortus*-infected alveolar epithelial cells was reduced by pretreatment with a specific caspase-1 inhibitor. This study shows that IL-1R, probably through IL-1β action, and the NLRP3 and AIM2 inflammasomes are involved in pulmonary innate immune protective mechanisms against respiratory *B. abortus* infection.

## Introduction

Brucellosis is a worldwide-distributed zoonotic disease caused by *Brucella* species, mainly *B. melitensis, B. suis*, and *B. abortus*, that affects over 500,000 people annually (Pappas et al., 2006). Inhalation of infected aerosols is a frequent way to acquire the infection in humans. Outbreaks of human brucellosis linked to airborne transmission have been reported in bovine and porcine slaughterhouses, laboratories producing *Brucella* vaccines, and rural areas (Hendricks et al., 1962; Kaufmann et al., 1980; Staszkiewicz et al., 1991; Wallach et al., 1997). Laboratory-acquired brucellosis, one of the most frequent laboratory-acquired infections (Yagupsky and Baron, 2005), has been mostly linked to aerosol transmission. Notably, CDC and NIAID have classified *Brucella* species as category B bioterrorism agents due to their easy aerosolization and high infectivity by the respiratory route (Pappas et al., 2006).

Interleukin-1 beta (IL-1β) has a central role in the early pulmonary immune response to inhaled pathogens, mainly due to its ability to induce the expression of several chemokines and adhesion molecules, to enhance the phagocytic activity of neutrophils and monocytic cells, and to increase the production of reactive oxygen species (Pinkerton et al., 2017). *In vivo* studies have shown that IL-1β produced by alveolar macrophages in response to *Legionella pneumophila* induces the secretion of neutrophil chemoattractants in lung epithelial cells, and similar results were obtained for *Streptococcus pneumoniae* infections (LeibundGut-Landmann et al., 2011; Marriott et al., 2012). IL-1β is produced as an inactive propeptide (pro-IL-1β) that needs to be processed in order to be secreted from activated monocytes, macrophages, and other cell types. The cleavage of pro-IL-1β into IL-1β is mediated by caspase-1, which is produced as pro-caspase-1 but matures into an active form after recruitment into multiprotein complexes known as inflammasomes (Lamkanfi and Dixit, 2012). These cytosolic complexes include caspase-1 and a sensor component responsible for detecting microbial components or cellular damage, and in some cases also include an adaptor molecule that serves to connect the first two. The sensor components of inflammasomes belong to the NOD-like receptor family (NLRP3, NLRC4, etc) or the HIN200 family (AIM2) of pattern recognition receptors. Therefore, upon activation due to recognition of microbial PAMPs or endogenous DAMPs, inflammasomes mediate the proteolytic cleavage of pro-IL-1β, thus generating mature IL-1β that can be secreted.

Several studies have shown the importance of inflammasomes for controlling bacterial infections, including those acquired by the respiratory route. Mice deficient in NLRC4 have a reduced survival to the intranasal infection with *Klebsiella pneumoniae* or *Legionella pneumophila* (Pereira et al., 2011; Cai et al., 2012), and those deficient in NLRP3 have higher mortality upon respiratory infection with *Streptococcus pneumonia* (Witzenrath et al., 2011). Similarly, mice deficient in AIM2 are highly susceptible to the intratracheal infection with *Mycobacterium tuberculosis* (Saiga et al., 2012). The expression of inflammasome components has been detected in several cell types from the respiratory system, including alveolar macrophages, bronchial and alveolar epithelial cells as well as endothelial cells (Cai et al., 2012; Hirota et al., 2012; Tran et al., 2012; Rotta detto Loria et al., 2013; Wu et al., 2013).

In spite of the importance of the respiratory route in brucellosis, the role of inflammasomes in protection against respiratory *Brucella* infection has not been studied. Here we show that caspase-1, NLRP3, and AIM2 are involved in the innate immune protection against *B. abortus* infection acquired through the respiratory route.

## Materials and methods

### Ethics statement

Animal experimentation was conducted in agreement with international ethical standards (Helsinki Declaration and its amendments, Amsterdam Protocol of welfare and animal protection, and National Institutes of Health, USA, guidelines: Guide for the Care and Use of Laboratory Animals). All animal experiments were preapproved by the Institutional Animal Care and Use Committee of UFMG (CETEA#128/2014).

### Mice

Wild-type C57BL/6 mice (6–9 wk of age) were purchased from the Federal University of Minas Gerais (UFMG), Brazil. Knock-out (KO) mice bred on C57BL/6 background (NLRP3, AIM2, caspase-1/11, and IL-1R KO mice) were provided by UFMG and have been described previously (Lara-Tejero et al., 2006; Rathinam et al., 2010; Vandanmagsar et al., 2011). All the strains of mice were housed in the same vivarium under the same conditions, and all received the same food and water sources. Animals were housed in groups of 5 animals, under controlled temperature (22 ± 2°C) and artificial light set to a 12 h cycle period. Mice were kept under specific pathogen-free conditions in positive-pressure cabinets and received sterile food and water *ad libitum*.

### Bacterial strains and growth conditions

*B. abortus* 2308 were grown to an OD_600_≈1.0 in tryptic soy broth (TSB) at 37 °C with agitation. After two washes with sterile phosphate buffered saline (PBS), bacteria were resuspended in sterile PBS to the desired OD_600_ to prepare inocula. All *Brucella* manipulations, including animal experiments, were conducted under BSL3 conditions. The involved personnel wore appropriate protection garment, including laboratory coats, gloves, and protective eyewear. These persons were trained on BSL3 practices before participation in the described experiments.

### Intratracheal inoculation

Animals were inoculated intratracheally with *B. abortus* as previously described (Revelli et al., 2012) with minor modifications. Briefly, mice received anesthesia with isoflurane and, after becoming recumbent, were injected with a mixture of ketamine and xylazine (100 and 8mg/kg) through the intraperitoneal route. Mice were placed in supine position over an acrylic backboard and restrained by the teeth using a rubber band. Under translucent illumination of the trachea, the inoculum (10^6^ colony forming units -CFU-per mice as estimated by OD_600_, 20 μl/mice) was injected in between the vocal cords with a Hamilton syringe coupled to a blunt-ended probe. In control mice 20 μl of PBS were inoculated following the same procedure. To check for efficient intratracheal inoculation and actual number of viable bacteria delivered, two sentinel animals were infected with *B. abortus* in each experiment, and were sacrificed at 2 h p.i. to measure CFU numbers in lung homogenates (mean values were 1.9 ± 0.3 × 10^5^ CFU per animal).

### CFU and cytokine analysis

Mice were euthanized at different time-points post-infection (p.i.) by injecting a lethal dose of ketamine and xylazine through the intraperitoneal route. Their lungs and spleens were harvested and homogenized in 2 ml of sterile PBS using a tissue homogeneizer with a 5 mm generator probe (Bio-Gen PRO200, PRO Scientific Inc., Oxford, CT), and serial dilutions of homogenate aliquots were plated on TSA for CFU counting. The remaining homogenate volumes were centrifuged for 15 min at 600x*g*, and the supernatants were mixed with protease inhibitors (cOmplete^TM^, Roche) and stored at −70°C. IL-1β and CXCL1 (KC) were measured in these samples using commercial ELISA kits, according to manufacturer's instructions. Briefly, IL-1β was measured using a sandwich ELISA (OptEIA, BD Biosciences, San Diego, CA) that included a monoclonal antibody for capture and a biotinylated monoclonal antibody for detection. CXCL1 was measured using a sandwich ELISA (DuoSet, R&D Systems, Minneapolis, MN) that included a monoclonal rat antibody for capture and a polyclonal goat biotinylated antibody for detection. In both cases the reaction was developed by a short incubation (20 or 30 min) with streptavidin-horseradish peroxidase conjugate followed by a washing step and the addition of a mixture of tetramethylbenzidine and hydrogen peroxide as the color reagent. The reactions were stopped with 2N H_2_SO_4_ and the resulting optical densities were read at 450 nm in an ELISA reader.

### Murine alveolar macrophages and broncho-alveolar lavage fluid (BALF)

Murine alveolar macrophages were isolated as previously described (Ferrero et al., 2014). Briefly, after mice were euthanized with a lethal dose of ketamine and xylazine injected through the intraperitoneal route, a fine-tipped pipette was inserted in their tracheas. Through this pipette the airways were perfused with sterile cold PBS containing 1 mM EDTA (3 perfusions, 1 ml each) to provide broncho-alveolar lavage fluid (BALF). After centrifugation at 400 x*g* for 10 min at 4°C, the supernatants were stored at −70°C for cytokine measurements (in the case of infected animals), whereas the cells present in the pellet were resuspended in complete medium (RPMI 1640 supplemented with 10 % heat inactivated fetal bovine serum -FBS, Gibco-BRL Life Technologies, Grand Island, NY-, 100 U of penicillin per ml, and 50 μg of streptomycin per ml). Cell viability as determined by trypan blue exclusion was routinely >95%. Cells were dispensed in 48-wells culture plates at 2 × 10^5^ cells/well, and were incubated for 2 h (37°C, 5% CO_2_ humidified atmosphere) to allow adhesion. After incubation cells were washed several times with fresh culture medium to remove non-adherent cells. Flow cytometry using anti-CD11c and anti-F4/80 antibodies was used to confirm that adherent cells were alveolar macrophages.

#### Primary alveolar type II (ATII) epithelial cells

ATII cells were isolated from murine lungs following the method described by Murphy et al. (Murphy et al., 1999). Lungs were perfused through the heart with sterile PBS to eliminate the remaining blood, and were then washed in solution A (0.130 M NaCl, 5.2 mM KCl, 10.6 mM Hepes, 2.6 mM Na_2_HPO_4_, 10 mM D-glucose, pH 7.4). Lungs were chopped to small pieces (< 0.5 mm thick), and the tissue was digested with trypsin (0.5% in solution A plus 1.9 mM CaCl_2_ and 1.29 mM MgSO_4_) by incubating at 37°C for 10 min in a water bath. The mixture was filtered through a 100 μm mesh, and the filtrate was recovered on FBS containing DNAse (Sigma; 400 U/ml). The resulting cellular suspension was filtered through a 40 μm mesh. The filtrate, containing individual cells, was centrifuged and the pellet enriched in ATII cells was resuspended in DMEM/F12 containing DNAse I (350 U/ml). The cellular suspension was seeded on 6-well culture plates to allow adhesion of contaminating leukocytes and other cells. Two hours later, non-adherent ATII cells were isolated from the supernatant using a discontinuous Percoll gradient (1.089 and 1.04 g/l; Sigma). The interface between the two Percoll layers, enriched in ATII cells, was recovered and washed twice with solution A. An aliquot was taken for Trypan blue staining and counting in a Neubauer chamber. For infection experiments, ATII cells were seeded at 2 × 10^5^ cells/well in DMEM/F12 supplemented with 10% FBS, 100 U/ml penicillin and 100 ug/ml streptomycin in 48-well plates coated with type I collagen. Cells were allowed to adhere for at least 36 h, non-adherent cells were discarded, and culture medium was changed every 24 h until confluence was reached (4–5 days).

#### Cell infections

Cells were infected at multiplicities of infection (MOI) of 100 bacteria/cell (AM) or 200 bacteria/cell (epithelial cells) in culture medium without antibiotics. After dispensing the bacteria the culture plates were centrifuged (10 min at 300 x*g* at room temperature) and then incubated for 2 h (37°C, 5% CO_2_ atmosphere). At the end of incubation (time 0 p.i.), the cells were washed three times with sterile PBS. Non-internalized bacteria were killed by incubating the infected cells with culture medium containing 100 μg/ml of gentamicin (Sigma, USA) and 50 μg/ml of streptomycin (Sigma, USA). At 24 h after antibiotics addition culture supernatants were harvested for cytokine measurement, whereas cells were washed with sterile PBS and lysed with 0.2% Triton X100. Serial dilutions of the lysates were plated on TSA to enumerate CFU of intracellular bacteria. In some experiments, cells were pretreated for 24 h with a specific caspase-1 inhibitor (Z-WEHD-FMK, R&D Systems, 5 μM) or its vehicle (DMSO) before infection, and the inhibitor was maintained during the whole infection period (Deepe and Buesing, 2012).

### Statistical analysis

Statistical comparisons for significant differences were performed with ANOVA followed by the appropriate *post-hoc* tests using GraphPad 5.0 software. Normality was assessed by the D'Agostino-Pearson test. Data are presented as means ± *SD* from three independent experiments. A *p* < 0.05 was considered as statistically significant.

## Results

### IL-1β is induced in lungs during *B. abortus* infection and IL-1R is involved in infection control

**Figure 1:**
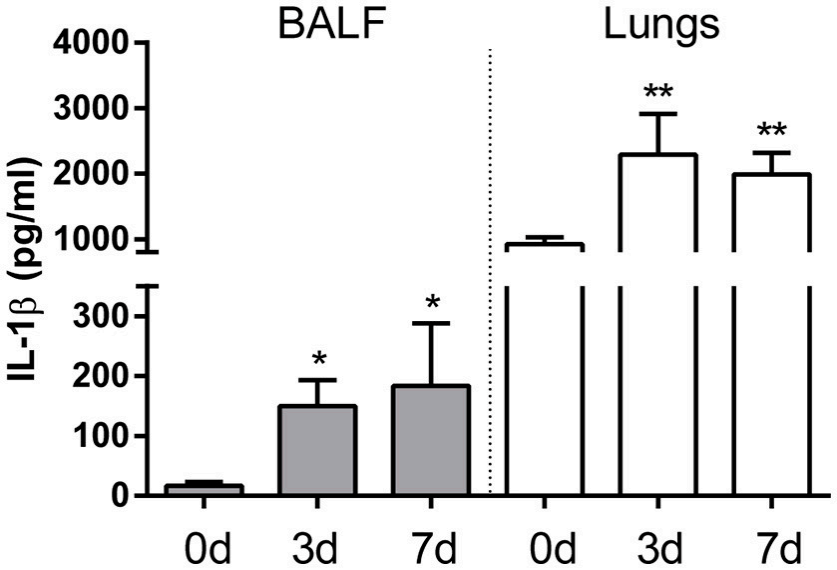
IL-1β is induced in lungs after intratracheal *B. abortus* infection. C57BL/6 mice were intratracheally infected with 10^6^ CFU/mouse of *B. abortus* in 20 μl of PBS. Mice (*n* = 5 per time point) were sacrificed before infection or at 3 and 7 days post-infection (0, 3, and 7d, respectively), and IL-1β was quantified in BALF (gray bars) and lung homogenates (white bars) of individual mice. Values are means ± SD of three independent experiments (^*^p < 0.05, ^**^p < 0.01 vs. basal levels, ANOVA followed by Dunnett's test).

To establish whether IL-1β production is induced during pulmonary *Brucella* infection, mice were intratracheally inoculated with *B. abortus*, and IL-1β levels were measured in lung homogenates and BALF at different times p.i. As shown in Figure 1, in both types of samples IL-1β levels were significantly higher at 3 and 7 days p.i. compared to basal levels.

**Figure 2:**
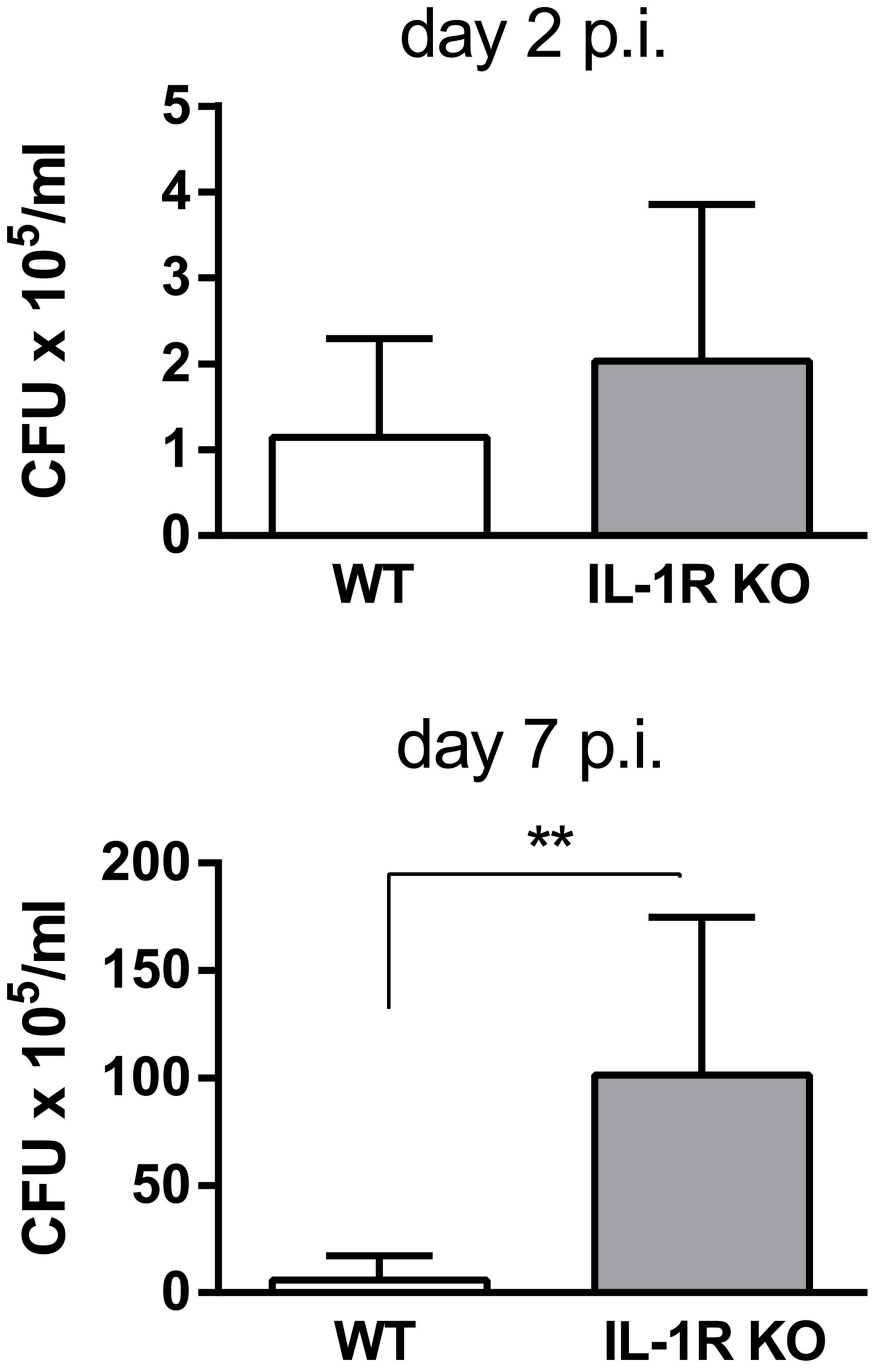
IL-1R is involved in the control of *B. abortus* replication in the lungs. C57BL/6 WT and IL-1R KO mice were intratracheally infected with 10^6^ CFU/mouse of *B. abortus* in 20 μl of PBS. At 2 and 7 days post-infection mice were sacrificed and CFU numbers were determined in lung homogenates (*n* = 5 mice per group per time). Values are means ± *SD* of three independent experiments (^**^p < 0.01, WT vs. IL-1R KO mice at each time p.i, Student's *t*-test).

To determine whether the IL-1β receptor (IL-1R) is important for the control of pulmonary *Brucella* infection, mice lacking this receptor were infected with *B. abortus* through the intratracheal route, and CFU were measured in lung homogenates obtained at different times p.i. As shown in Figure 2, CFU were significantly higher in IL-1R KO mice than in wild type (WT) mice at 7 days p.i.

### Inflammasomes are involved in the protection against pulmonary *B. abortus* infection

**Figure 3:**
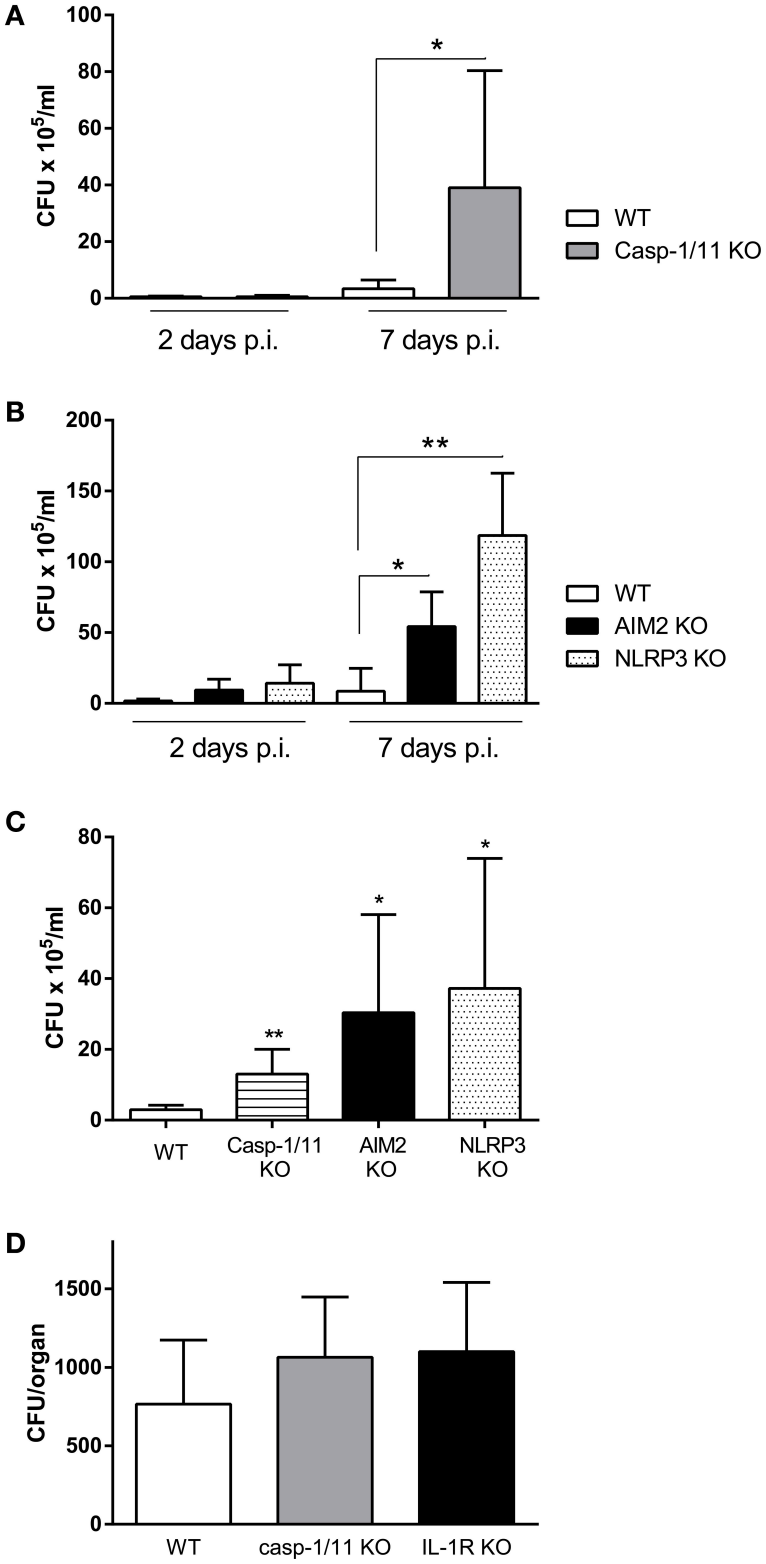
Inflammasomes are involved in the control of *B. abortus* replication in the lungs. C57BL/6 WT mice or knock-out (KO) mice for caspase-1 (Casp 1/11 KO) were infected intratracheally with *B. abortus*, and CFU numbers were determined in lung homogenates at days 2 and 7 p.i. **(A)**. A similar experiment was performed with WT mice and KO mice for AIM2 and NLRP3 **(B)**. WT mice and KO mice for the three inflammasome components were infected as above and CFU were determined in BALF at day 3 p.i. **(C)**. Spleens were harvested from IL-1R KO and caspase-1/11 KO mice (and WT controls) at 7 days after intratracheal infection with *B. abortus* and were processed for CFU counting **(D)**. In all the experiments, *n* = 5 mice per group per time point. Values are means ± *SD* of three independent experiments (^*^p < 0.05, ^**^p < 0.01, ^***^p < 0.001; WT vs. KO for the same day p.i., ANOVA followed by Tukey's test).

IL-1β is produced as a precursor by different cell types, and it requires to be cleaved to mature IL-1β for secretion, being inflammasomes, through caspase-1 activity, the most important mediators of such cleavage. To determine whether inflammasomes are important for protection against respiratory *Brucella* infection, mice lacking caspase-1 were intratracheally infected with *B. abortus*, and CFU were determined in pulmonary samples at different times p.i. As shown in Figure 3A, CFU levels in lungs were higher in caspase-1/11 KO mice as compared to WT controls. As previous studies have shown that AIM2 and NLRP3 are important for protection against intraperitoneal *B. abortus* infection (Gomes et al., 2013), the relevance of these molecules for protection against respiratory *B. abortus* infection was tested. As shown in Figure 3B, CFU levels in lungs were higher in AIM2 KO and NLRP3 KO mice as compared to WT controls. Similar differences between KO mice for inflammasome components and WT mice were observed when BALF samples were analyzed (Figure 3C). To determine whether IL-1R and inflammasomes may also influence the systemic dissemination of *B. abortus* after respiratory infection, spleens were harvested from IL-1R KO and caspase-1/11 KO mice (and WT controls) at 7 days p.i. and were processed for CFU counting. As shown in Figure 3D, CFU tended to be higher in KO mice than in WT mice, although the difference did not reach statistical significance.

### Pulmonary levels of IL-1β and CXCL1 are reduced in caspase-1/11 KO mice

**Figure 4:**
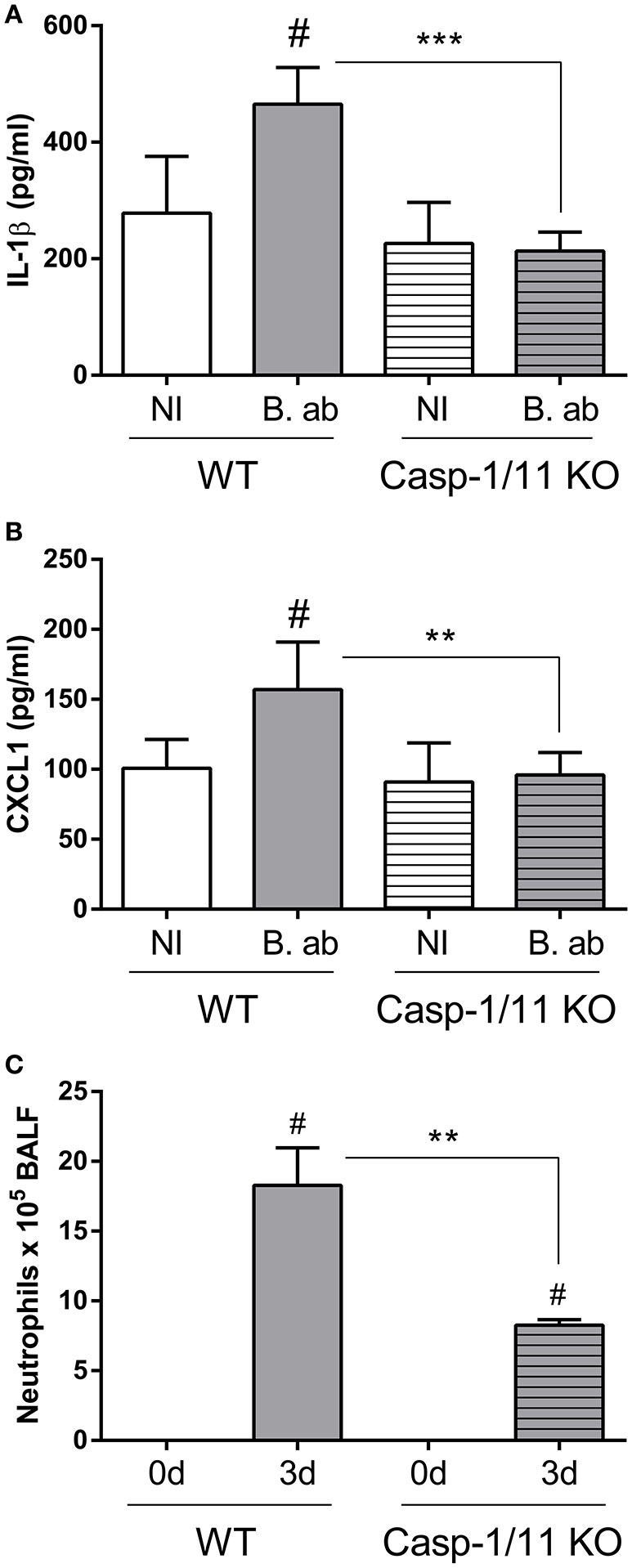
Initial lung IL-1β and CXCL1 production upon *B. abortus* infection is dependent on caspase-1 expression. Groups of C57BL/6 WT and caspase-1/11 KO mice were intratracheally infected with 10^6^ CFU/mouse of *B. abortus* in 20 μl of PBS (B. ab, gray bars) or administered with 20 μl of PBS alone (NI, non-infected controls, white bars). At 2 days post-infection IL-1β **(A)** and CXCL1 **(B)** levels were quantified in lung homogenates by ELISA (*n* = 5 mice per group per treatment). **(C)** Groups of C57BL/6 WT and caspase-1/11 KO mice were intratracheally infected as described, and neutrophils counts were determined in BALF before infection (0d) and at 3 days p.i. (*n* = 5 mice per strain per time point). Values are means ± *SD* of three independent experiments (# *p* < 0.05, NI vs. infected within mice strains, Student's *t*-test; ^**^p < 0.01, ^***^p < 0.001, WT vs. caspase-1 KO, Student's *t*-test).

The importance of caspase-1 for controlling *B. abortus* infection in lungs, as shown above, may be related to its ability to mediate IL-1β maturation, allowing its secretion to the external milieu. Therefore, experiments were performed to test whether caspase-1/11 KO mice have a reduced IL-1β response to pulmonary *B. abortus* infection. As shown in Figure 4A, at 2 days p.i. IL-1β levels were significantly lower in KO mice as compared to WT controls. As IL-1β is known to induce the expression of chemokines in lung cells (Crestani et al., 1994; Koyama et al., 1999; Pechkovsky et al., 2000; Ishii et al., 2005), the levels of CXCL1 (also known as KC), a murine chemoattractant for neutrophils, were also measured. As shown in Figure 4B, CXCL1 levels were also significantly reduced in caspase-1/11 KO mice at 2 days p.i. In line with these findings, while neutrophils were recruited to the airways in response to *B. abortus* infection in both strains of mice, the number of neutrophils in BALF at 3 days p.i. was significantly lower in caspase-1/11 KO mice as compared to WT controls (Figure 4C).

### NLRP3 and AIM2 inflammasomes are involved in IL-1β production by alveolar macrophages in response to *B. abortus* infection

**Figure 5:**
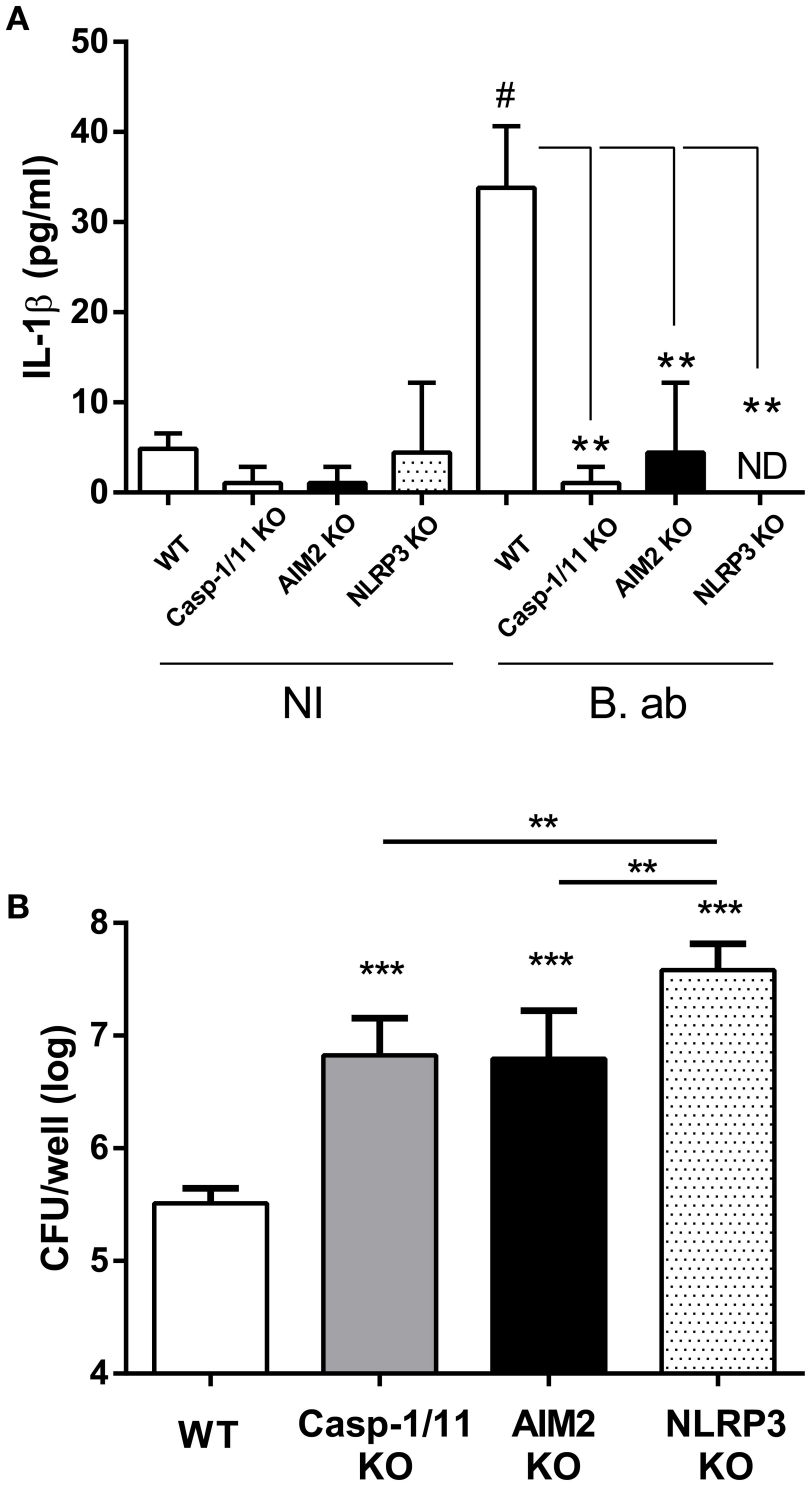
IL-1β produced by alveolar macrophages (AM) upon *B. abortus* infection is dependent on AIM2 and NLRP3 inflammasome expression. AM were obtained from C57BL/6 WT and caspase-1/11, AIM2 and NLRP3 KO mice. Cells were infected with *B. abortus* (B. ab) at a MOI of 100 or were left uninfected (NI, control group). IL-1β levels were measured in culture supernatants obtained at 17 h p.i. (triplicates per group and treatment) **(A)**. The infected AM were washed and lysed, and lysates were plated for the determination of viable intracellular bacteria **(B)**. Values are means ± *SD* of three independent experiments (# *p* < 0.05, NI vs. B. ab groups within each mice strain, Student's *t*-test; ^***^p < 0.001 KO vs. WT, ^**^p < 0.01 ANOVA followed by Tukey's test).

Alveolar macrophages (AM) are important sources of IL-1β within the lungs (31, 32). To analyze the importance of inflammasomes for IL-1β production by AM during *B. abortus* infection, these cells were obtained from WT C57BL/6 mice (controls) or from mice deficient in caspase-1, NLRP3 or AIM2 (KO mice), and were infected *in vitro* with this pathogen. IL-1β levels were measured in culture supernatants at 17 h p.i. As shown in Figure 5A, IL-1β secretion was significantly reduced in AM from all the three strains of mice deficient in inflammasome components as compared to control mice. In addition, AM obtained from the three KO strains had a reduced microbicidal activity against *B. abortus* as compared to those obtained from the WT strain (Figure 5B).

**Figure 6:**
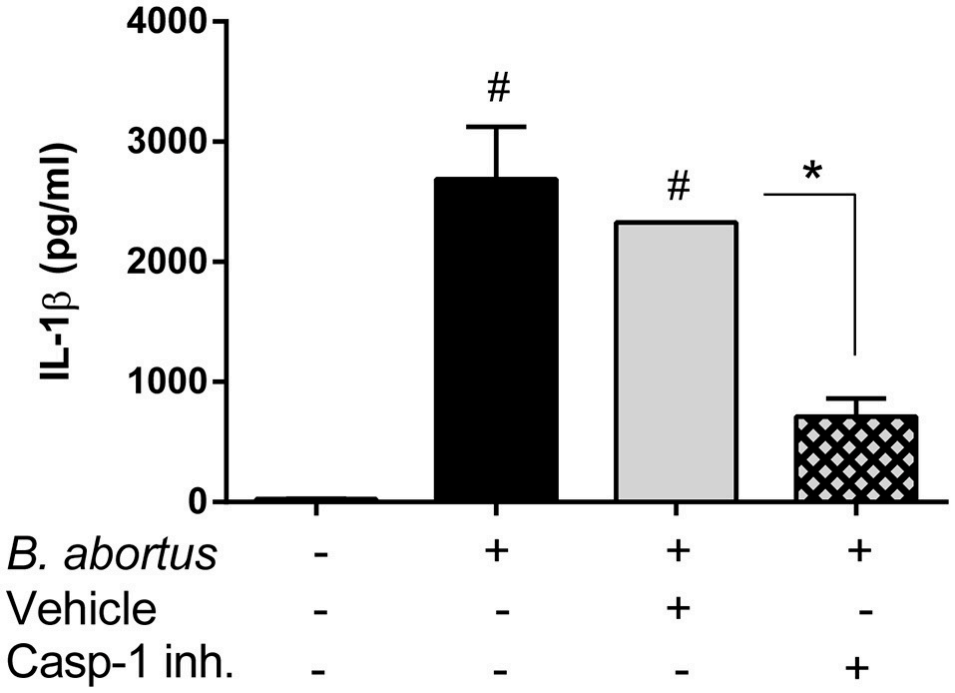
Production of IL-1β by alveolar epithelial cells upon *B. abortus* infection is dependent on caspase-1 activity. Alveolar epithelial cells were obtained from C57BL/6 WT mice and seeded at a density of 2 × 10^5^ cells/well. Cells were infected, or not, with *B. abortus* and treated either with caspase-1 inhibitor (Z-WEHD-FMK, 5 μM) or the equivalent amount of vehicle (DMSO). IL-1β levels were quantified in culture supernatants obtained at 24 h p.i (duplicates per group and treatment). Values are means ± *SD* of three independent experiments (#*p* < 0.05, non-infected vs. *B. abortus* infected groups, ANOVA followed by Dunnett's test; ^*^p < 0.05, vehicle vs. inhibitor treatment, Student's *t*-test).

In addition to AM, alveolar epithelial cells also produce IL-1β upon stimulation with microbial or environmental antigens (Wang et al., 2002; Thorley et al., 2007). To test whether inflammasomes are also important for IL-1β production by alveolar epithelial cells during *B. abortus* infection, primary cultures of these cells (type II pneumocytes or ATII) obtained from WT mice were treated with a specific caspase-1 inhibitor before infection with the pathogen, and IL-1β levels were measured at 24 h p.i. As shown in Figure 6, IL-1β levels were significantly reduced in cells pretreated with the inhibitor as compared to untreated cells or cells treated with vehicle (DMSO).

## Discussion

Inhalation of infected aerosols is one of the most frequent ways to acquire *Brucella* spp. infection, and airborne transmission has been linked to outbreaks of human brucellosis in different settings (Hendricks et al., 1962; Kaufmann et al., 1980; Staszkiewicz et al., 1991; Wallach et al., 1997; Yagupsky and Baron, 2005; Pappas et al., 2006). In spite of the importance of the respiratory route for this disease, the pulmonary immune response to *Brucella* spp. acquired through the airways has only recently been addressed by some studies (Surendran et al., 2012; Hanot Mambres et al., 2016; Hielpos et al., 2017). Until now, however, the potential role of inflammasomes in the innate pulmonary response to this pathogen has not been evaluated.

Inflammasomes are multiprotein structures whose activity is essential for the maturation and secretion of IL-1β and IL-18. In particular, IL-1β is known to have a central role in the pulmonary immune response to inhaled pathogens during the first days after infection, which was the time frame addressed in the present study. Notably, we found that IL-1β levels were significantly increased in both lung homogenates and BALF of mice at 3 and 7 days after intratracheal infection with *B. abortus*. These findings agree with those of a previous study using a similar infection protocol (Hielpos et al., 2017). IL-1β may also contribute to the control of *B. abortus* infection in the lungs, as mice lacking IL-1R exhibited a significant increase in CFU counts in lung homogenates at 7 days p.i. compared to control mice. This hypothesis is supported by the results obtained regarding inflammasomes involvement, as discussed below. The importance of IL-1β for controlling *B. abortus* infection in lungs may be related to one or more of its known actions, including the induction of the expression of chemokines and adhesion molecules, the promotion of the phagocytic activity of neutrophils and monocytic cells, and the stimulation of reactive oxygen species production (Pinkerton et al., 2017). In some infections by airborne pathogens, IL-1β produced by alveolar macrophages has been shown to induce the secretion of neutrophils chemoattractants by lung epithelial cells (LeibundGut-Landmann et al., 2011; Marriott et al., 2012). A previous study on intranasal *B. melitensis* infection did not find increased CFU counts in IL-1R KO mice (Hanot Mambres et al., 2016). It must be noted, however, that apart from differences in *Brucella* species and infection route when compared to the present study, the infecting dose used in that previous study was significantly lower (2 × 10^4^ CFU/mice).

IL-1β is synthesized as a precursor (pro-IL-1β) by different cell types, and it must be cleaved to mature IL-1β for secretion. Inflammasomes, which are multimeric structures composed of at least caspase-1 and a sensor component (AIM2 or a member of the NLR family), are the most important mediators of pro-IL-1β cleavage (Lamkanfi and Dixit, 2012). In the present study we show that some inflammasomes are involved in the protection against pulmonary *B. abortus*. After intratracheal infection with this pathogen, CFU counts in lungs and/or BALF were higher in mice lacking caspase-1, AIM2 or NLRP3 compared to C57BL/6 controls. Of note, these results are in line with those of a previous study that showed that caspase-1, AIM2 and NLRP3 are important for protection against intraperitoneal *B. abortus* infection (Gomes et al., 2013). A plausible explanation for the impaired control of pulmonary *B. abortus* infection in mice lacking inflammasome components may be a reduction in the secretion of IL-1β, which, as shown in this study, is important for such control. In line with this hypothesis, pulmonary levels of IL-1β at 2 days p.i. were found to be reduced in caspase-1/11 KO mice when compared to control mice. IL-1β has many actions that could potentially contribute to the control of *B. abortus* infection in the lungs, including the induction of chemokines, antimicrobial peptides, adhesion molecules, etc (Koyama et al., 1999; Pechkovsky et al., 2000; Ishii et al., 2005). In this study, caspase-1/11 KO mice had reduced pulmonary levels of KC (CXCL1, an important neutrophil chemoattractant), and also exhibited a significantly lower number of neutrophils in BALF after infection. This suggests that the reduced production of chemokines relevant for the recruitment of phagocytes may be one of the factors that contribute to the impaired control of the pulmonary *B. abortus* infection in these mice.

After respiratory infection in mice *Brucella* spp. disseminates systemically and colonizes several target organs, particularly the spleen (Surendran et al., 2012; Hanot Mambres et al., 2016; Hielpos et al., 2017). As commented above, the pulmonary CFU levels of *B. abortus* were increased in mice lacking IL-1R or inflammasome components. Interestingly, CFU in spleen also tended to be higher in IL-1R KO and caspase-1/11 KO mice than in WT mice. Although the difference did not reach statistical significance, probably due to the early p.i. time of study (7 days p.i.), it suggests that the impaired immune control of *B. abortus* infection at its pulmonary portal of entry may translate into increased numbers of bacteria reaching peripheral organs. Alternatively, or in addition, this higher bacterial load may be due to a reduced immune control of the pathogen at these organs in KO mice.

Within the lungs, alveolar macrophages (AM) and alveolar epithelial cells are among the most important sources of IL-1β production after an antigenic or microbial insult (Losa García et al., 1999; Wang et al., 2002; Malazdrewich et al., 2004; Thorley et al., 2007). Our results not only showed that both cells types produce IL-1β in response to *B. abortus* infection, but also that such production depends on the activity of inflammasomes. Although IL-1β production by AM from control mice was modest, in agreement with previous studies (Ferrero et al., 2014), it was significantly reduced in AM from mice deficient in inflammasome components (caspase-1, NLRP3 or AIM2). Similarly, IL-1β levels were significantly reduced in primary pneumocytes pretreated with a specific caspase-1 inhibitor as compared to untreated cells or cells treated with vehicle. In addition, AM obtained from KO mice for inflammasome components also exhibited a reduced microbicidal activity against *B. abortus* as compared to those obtained from the WT strain. Further studies will be required to disclose the mechanisms underlying this impairment.

In summary, the present study demonstrated that inflammasomes are important for IL-1β production in the lungs after respiratory *Brucella* infection, and to restrain the development of the pathogen in the pulmonary tissue. Alveolar macrophages and pneumocytes seem to contribute to IL-1β production in the context of *B. abortus* infection, in an inflammasome-dependent manner. Overall, inflammasomes are important for the control of pulmonary *B. abortus* infection.

## Author contributions

MF, SO, and PB conceived and designed the experiments. MH, MF, AF, IA, FM, PC, AV, and JF performed the experiments. MH, MF, AF, PC, AV, JF, SO, and PB analyzed the data; MF, SO, and PB wrote the draft and/or final version of the paper. All authors reviewed the manuscript.

### Conflict of interest statement

The authors declare that the research was conducted in the absence of any commercial or financial relationships that could be construed as a potential conflict of interest.
